# The diversity of stomatal development regulation in *Callitriche* is related to the intrageneric diversity in lifestyles

**DOI:** 10.1073/pnas.2026351118

**Published:** 2021-03-29

**Authors:** Yuki Doll, Hiroyuki Koga, Hirokazu Tsukaya

**Affiliations:** ^a^Graduate School of Science, The University of Tokyo, Tokyo 113-0033, Japan

**Keywords:** stomatal development, amplifying division, *Callitriche*, amphibious plants, evodevo

## Abstract

Stomata, the gas exchange structures of plants, are formed by the division and differentiation of stem cells, or meristemoids. Although diverse patterns of meristemoid behavior have been observed among different lineages of land plants, the ecological significance and diversification processes of these different patterns are not well understood. Here we describe an intrageneric diversity in the patterns of meristemoid division within the ecologically diverse genus *Callitriche* (Plantaginaceae). Meristemoids underwent a series of divisions before differentiating into stomata in the terrestrial species of *Callitriche*, but these divisions did not occur in amphibious species, which can grow in both air and water, in which meristemoids differentiated directly into stomata. These findings imply the adaptive significance of diversity in meristemoid division. Molecular genetic analyses showed that the different expression times of the stomatal key transcription factors SPEECHLESS and MUTE, which maintain and terminate the meristemoid division, respectively, underlie the different division patterns of meristemoids. Unlike terrestrial species, amphibious species prematurely expressed *MUTE* immediately after expressing *SPEECHLESS*, which corresponded to their early termination of stomatal division. By linking morphological, ecological, and genetic elements of stomatal development, this study provides significant insight that should aid ecological evolutionary developmental biology investigations of stomata.

## Significance

Plant stomata are produced through divisions and differentiation of stem cells, termed meristemoids. During stomatal development, we see diverse patterns of meristemoid behavior among land plant lineages. However, both the ecological significance and the diversification processes of this diversity remain mostly unknown. Here we report that the ecologically diverse genus *Callitriche* shows unprecedented intrageneric diversity in meristemoid behavior. While meristemoids in terrestrial species of *Callitriche* undergo a series of asymmetric divisions before differentiation, those in amphibious species skip the divisions and directly differentiate into stomata. The simple shift in the expression times of two key transcription factors underlies these different patterns. This study provides important insights into the evolution and ecological significance of stomatal patterning.

**Fig. 1.**
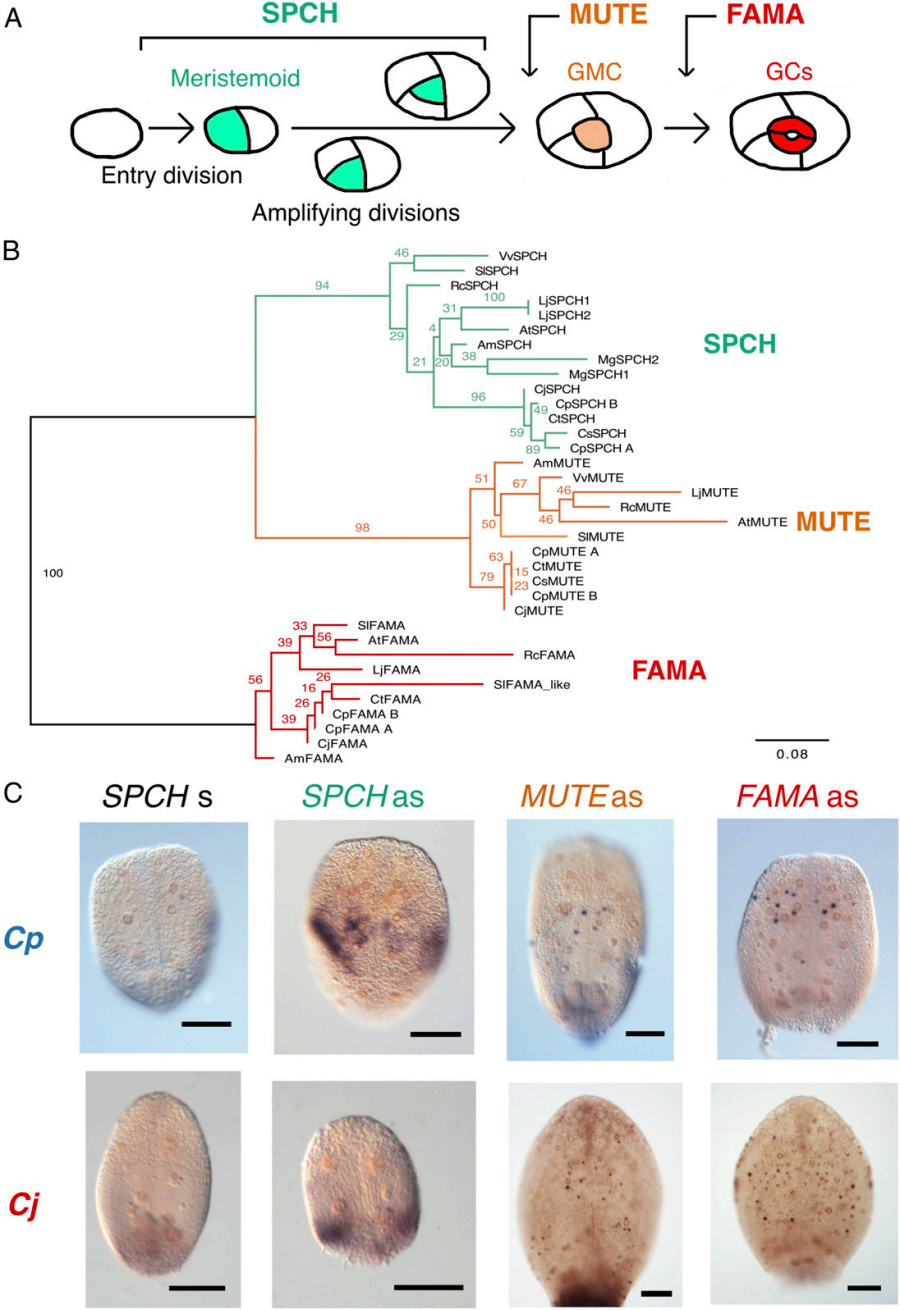
Identification of SMF orthologs in *Callitriche*. (*A*) Schematic showing the process of stomatal development and the roles of SMF proteins in Arabidopsis. (*B*) Phylogenetic tree of SMF proteins. Numbers at nodes indicate confidence values (%) calculated from 1,000 bootstraps. In addition to *Callitriche* species, sequences from the following taxa are included: *Arabidopsis* (At), tomato (*Solanum lycopersicum*; Sl), common grape vine (*Vitis vinifera*; Vv), castor bean (*Ricinus communis*; Rc), *Mimulus guttatus* (Mg), and *Lotus japonicus* (Lj). (*C*) WISH results for *SMF* orthologs in *C*. *palustris* and *C. japonica* leaf primordia. Results obtained using antisense (as) probes for each ortholog are shown, along with those using sense (s) probes for the *SPCH* orthologs. (Scale bars: 100 μm.)

The stoma is a plant gas exchange structure comprising a pair of kidney-shaped guard cells (GCs). The developmental process of GCs has been extensively studied in the model plant Arabidopsis. Previous studies identified three basic helix-loop-helix (bHLH) transcription factors that act as master regulators of stomatal development in this species (1–3) (Fig. 1*A*). Stomatal development begins in the polygonal stomatal stem cell, the meristemoid, which is produced from a protodermal cell by an asymmetric division (the entry division). A newly produced meristemoid undergoes a series of self-renewing asymmetric divisions (the amplifying divisions). The transcription factor SPEECHLESS (SPCH) is necessary to establish the meristemoid and for maintenance of subsequent amplifying divisions (3). The amplifying divisions are terminated by a second key transcription factor, MUTE, which promotes meristemoid differentiation into rounded guard mother cells (GMCs) (2). Finally, the transcription factor FAMA regulates the symmetric division of a GMC to produce a pair of GCs (1). These key transcription factors, collectively termed SMF (SPCH/MUTE/FAMA) proteins, form a single clade in the bHLH family and are broadly conserved in land plant lineages (4, 5). Recent studies have shown that SMF orthologs have conserved functions in the stomatal development of other model land plant species, such as rice (6), maize (6, 7), *Brachypodium distachyon* (8, 9), tomato (10), and *Physcomitrium patens* (11).

Stomata are of particular interest for evolutionary studies because of their ecological significance and phylogenetically diverse patterns of development (12–15). However, no study has characterized the diversification process at the species level, probably because molecular information in nonmodel species is lacking. The number and direction of meristemoid divisions differ among different taxa (12, 15–17). Different functions or activities of SPCH and MUTE have been proposed as mechanisms underlying this diversity in meristemoid behavior (12, 16), but no empirical study has explored the genetic basis for this.

A further example of diversity in stomatal development is found in amphibious plants, which can grow in both air and water. Amphibious plants have highly plastic development, in which they change their morphological and physiological phenotypes depending on whether they are submerged (18). Many amphibious plants have the ability to suppress stomatal development only when they are submerged. Although this plasticity in stomatal development has long been known for amphibious species from broad lineages (19–22), the molecular basis is still largely unexplored. To investigate this phenomenon, we previously described the process of leaf and stomatal development in the amphibious plant *Callitriche palustris* L. under highly reproducible conditions (23).

*Callitriche* (Plantaginaceae) is a cosmopolitan genus comprising 50 to 75 known species with diverse lifestyles that range from terrestrial to amphibious to completely aquatic (24). In addition to the existence of related species with diverse lifestyles, its diminutive stature and ease of laboratory culture make *Callitriche* an attractive model for eco-evolutionary investigations of diverse aspects of amphibious life in plants, including stomatal suppression induced by submergence. We recently described the dramatic suppression of stomatal development in submerged leaves of *C*. *palustris* (23). We also found that both aerial and submerged leaves of this species differentiate stomata during a restricted developmental period, when the leaf primordia are ∼1 mm long (23). This contrasts strongly with the well-described process in *Arabidopsis*, in which the number of stomata continues to increase in even later stages of leaf development (25). Further in-depth investigations are required to understand this unique pattern of stomatal development, which may be associated with the amphibious lifestyle and submergence-induced stomatal suppression.

In this study, we conducted comparative analyses within the genus *Callitriche* to better characterize the transient nature of stomatal development in *C*. *palustris*. We showed that both *C*. *palustris* and another amphibious *Callitriche* species lack meristemoid-amplifying divisions, leading to a sudden increase in the number of stomata within a restricted developmental period. More interestingly, we showed that terrestrial species of *Callitriche* have the normal amplifying divisions found in *Arabidopsis*. Such intrageneric diversity in meristemoid division among life forms has not been demonstrated previously and should serve as a useful model for evolutionary studies. We performed genetic analyses focusing on *SPCH* and *MUTE* to elucidate the diversification process of meristemoid division at the species level.

## Results

### Differing Patterns of Stomatal Development in Terrestrial and Amphibious Species of *Callitriche*.

**Fig. 2.**
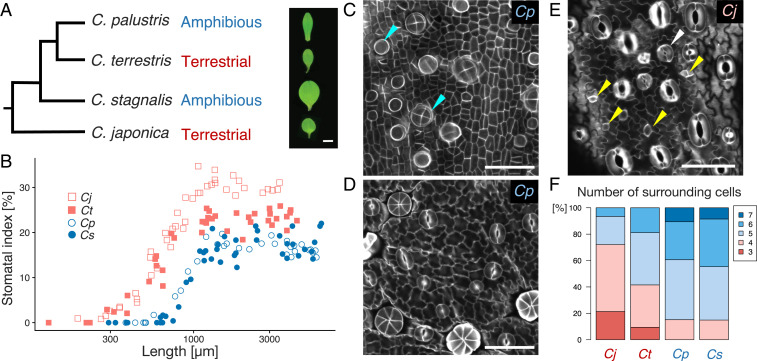
Species in the ecologically diverse genus *Callitriche* have diverse stomatal development patterns. (*A*) Phylogenetic tree of the species included in this investigation, based on previous studies (26, 27). The morphologies of single mature leaves of each species are shown on one side. (Scale bar: 1 mm.) (*B*) Quantification of the stomatal index throughout the developmental period. Each dot corresponds to one leaf primordium. (*C*–*E*) Confocal images of Calcofluor-stained developing leaf primordia of *C*. *palustris* (*C* and *D*, adaxial epidermis) and *C*. *japonica* (*E*, abaxial epidermis). Primordium lengths: 495 μm (*C*), 1,198 μm (*D*), and 889 μm (*E*). (Scale bars: 50 μm.) Yellow arrowheads in *E* indicate meristemoids undergoing amplifying divisions; the white arrow indicates a stoma surrounded by three cells that probably arose via amplifying divisions (see text). Radially arranged cell groups and rounded cells with strong Calcofluor signals, indicated by the blue arrowheads in *C*, are hair cells and their primordia, respectively. (*F*) Quantification of the number of pavement cells surrounding a stoma. For each species, at least 48 mature stomata were examined.

To identify unique temporal patterns of stomatal development in *C*. *palustris*, we also investigated the time course of stomatal development in three other *Callitriche* species with different lifestyles. In addition to *C*. *palustris*, we analyzed a second amphibious species from a different clade within the genus, *Callitriche stagnalis*. We also examined two terrestrial species, *Callitriche japonica* and *Callitriche terrestris*. *C*. *japonica* belongs to the ancestral clade, while *C*. *terrestris* is a more derived species that is phylogenetically closest to *C*. *palustris* among the three newly analyzed species (26, 27) (Fig. 2*A*). These differences among species allowed us to examine lifestyle-associated traits with minimal phylogenetic constraints.

Stomata were distributed at high densities on both surfaces of the leaves of *C*. *terrestris*, but the other species had biased adaxial-abaxial distributions of stomata (*SI Appendix*, Fig. S1*A*). *C*. *japonica* had more stomata on the abaxial side, and the amphibious species *C*. *palustris* and *C*. *stagnalis* had more stomata on the adaxial side. Thus, we focused on the abaxial epidermis of terrestrial species and the adaxial epidermis of amphibious species in further analyses. We observed the epidermis of developing leaf primordia using confocal microscopy and calculated the stomatal index over the course of leaf development (Fig. 2*B*). We found that, as reported previously, stomatal development in *C*. *palustris* occurred in restricted development phases (∼500 to 1,000 μm leaf primordium length), and also that this was the case in another amphibious species, *C*. *stagnalis*. In contrast, the terrestrial species started to develop stomata in much smaller leaf primordia (∼200 μm long). Terrestrial species developed stomata in more developmental stages, and the duration of stomatal development in terrestrial plants appeared to be longer compared with the amphibious species. This difference persisted even after normalizing the leaf primordium length by the maximum leaf length of each species (*SI Appendix*, Fig. S1*B*).

The two amphibious species had highly synchronous stomatal development; stomatal lineage cells in close proximity were all at similar developmental stages (Fig. 2 *C* and *D* and *SI Appendix*, Fig. S1*C*). In addition, we rarely identified polygonal meristemoid-like cells in these species. In both of the terrestrial species, on the other hand, we observed a wide spectrum of developmental stages in the stomatal lineage and found a number of polygonal meristemoid-like cells near the developed stomata (Fig. 2*E* and *SI Appendix*, Fig. S1 *D* and *E*). These observations imply that stomatal lineage cells in terrestrial species go through amplifying divisions before differentiating into GMCs, whereas those of amphibious species do not, which may explain the gradual increase in stomatal number in terrestrial species and the sudden increase in amphibious species.

Our analyses of epidermal cell arrangement provided another line of evidence supporting this postulate. Helical amplifying divisions successively produce three pavement cells and finally a pair of GCs in the center, forming a monoclonal “anisocytic complex” (16, 28) (Fig. 1*A*). We found that some mature stomata were surrounded by three pavement cells in terrestrial species, but not in amphibious species (Fig. 2 *E* and *F* and *SI Appendix*, Fig. S1*D*). The difference in the adaxial-abaxial distribution of stomata between terrestrial and amphibious species should be interpreted with caution (*SI Appendix*, Fig. S1*A*), because the pattern of stomatal development may be affected by the genetic system defining leaf polarity (29). Nevertheless, we did observe meristemoid-like cells in the adaxial epidermis of *C. terrestris* (*SI Appendix*, Fig. S1*E*), and the arrangement and developmental time course of stomata in the adaxial epidermis of *C. terrestris* were similar to those in the abaxial epidermis (*SI Appendix*, Fig. S1 *F* and *G*). Thus, we conclude that it is not the adaxial-abaxial stomatal distribution, but rather the lifestyle of each species, that is associated with the presence of amplifying divisions.

### Amphibious Species Lack Amplifying Divisions in the Stomatal Lineage.

**Fig. 3.**
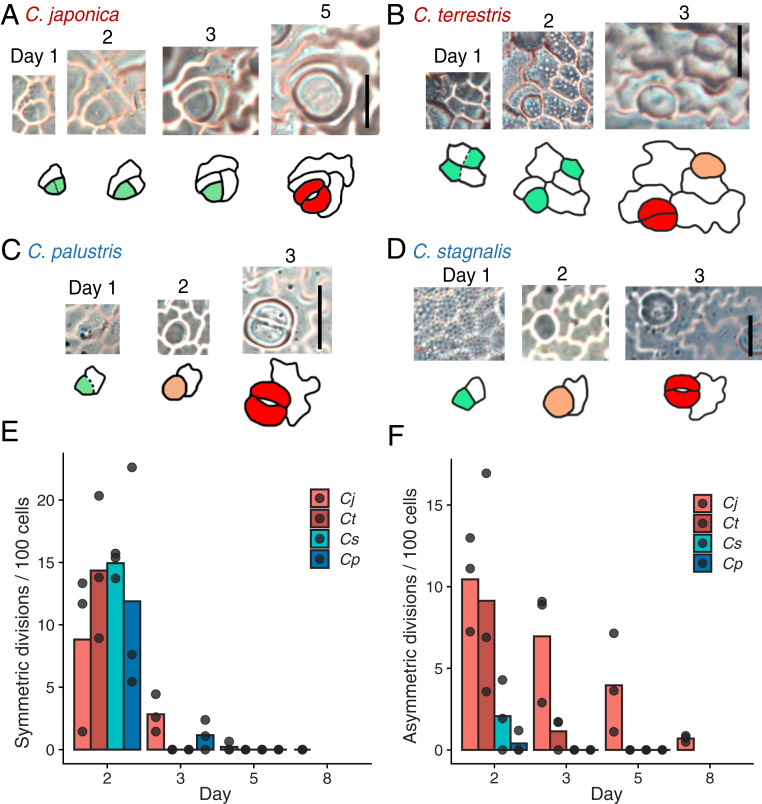
Meristemoids in terrestrial species undergo amplifying divisions, whereas those in amphibious species do not. (*A*–*D*) A series of epidermal impressions showing the process of stomatal differentiation in *C. japonica* (*A*), *C*. *terrestris* (*B*), *C*. *palustris* (*C*), and *C*. *stagnalis* (*D*). Dotted lines indicate a thin cell wall that was assumed to have been newly formed just before the impression was made. The colors in the traced images correspond to individual stomatal differentiation stages (Fig. 1*A*). (Scale bars: 20 μm.) (*E* and *F*) Cell division profile obtained by time-lapse analysis of epidermal impressions. Each dot indicates a result obtained from a single observed region (comprising >45 cells on the first day) in different leaf primordia from different individuals, reflecting a biological replicate. (*E*) Number of symmetric cell divisions outside the stomatal lineage. (*F*) Number of asymmetric cell divisions in the stomatal lineage.

To obtain direct evidence of the presence or absence of amplifying divisions in *Callitriche* species, we conducted time-lapse observations of the leaf epidermis through the construction of serial epidermal replicas using dental impression medium. As expected, we observed clear amplifying divisions before the differentiation of stomata in both terrestrial species (Fig. 3 *A* and *B* and *SI Appendix*, Fig. S2*B*). We did not find any amplifying divisions in the stomatal lineages in the two amphibious species; that is, all the meristemoids produced by entry division differentiated directly into GMCs without any additional asymmetric divisions (Fig. 3 *C* and *D* and *SI Appendix*, Fig. S2*A*). We cannot exclude the possibility that some stomata in amphibious species are produced by the direct establishment of GMCs from protodermal cells, as observed in the moss and hornwort species (11, 30), but the morphology of the earliest stomatal lineage cells had asymmetric polygonal shapes, even in the amphibious species (Fig. 3 *C* and *D*), which indicates that most of these cells were produced by an entry asymmetric division (Fig. 3*C*). Whereas both terrestrial and amphibious species had active cell divisions outside the stomatal lineage (Fig. 3*E*), only terrestrial species had prolonged asymmetric divisions in the stomatal lineage (Fig. 3*F*). Thus, the meristemoids in terrestrial species clearly underwent a series of amplifying divisions, but the meristemoids in amphibious species skipped this step and proceeded directly to differentiation. This discovery demonstrates a clear correlation between plant lifestyle and meristemoid behavior.

### Identification of SMF Orthologs in *Callitriche*.

We next aimed to elucidate the molecular basis for this diversity in the meristemoid division patterns in *Callitriche*. We focused on the *SMF* genes, which constitute the most fundamental component of the molecular pathways regulating stomatal development in *Arabidopsis* (1–3) (Fig. 1*A*) and, arguably, all land plant lineages (13). To identify putative *SMF* orthologs in *Callitriche*, we used the Basic Local Alignment Search Tool (BLAST) to compile amino acid sequences similar to the *AtSMFs* in our RNA-sequencing (RNA-Seq) datasets for *Callitriche* (31) and public databases (details in *Materials and Methods*). Based on the amino acid sequences collected, we constructed a maximum likelihood tree and found three monophyletic groups corresponding to each SMF protein (Fig. 1*B*). We identified two putative orthologs per *SMF* in *C*. *palustris*, and one putative ortholog in the other three species within each clade. Since previous karyotype analyses have indicated that *C*. *palustris* is a tetraploid and that *C*. *terrestris* and *C*. *stagnalis* are diploids (32, 33), the two orthologs in *C*. *palustris* are likely a pair of paralogs after genome duplication, and therefore we named them *CpSPCH A* and *CpSPCH B*, for example. Note that each putative paralogous gene in *C*. *palustris* was ≥90% identical in its amino acid sequence to the other paralog. Given this information and our RNA-Seq data showing that the expression patterns under various conditions were almost the same between paralogs (31) (*SI Appendix*, Fig. S3*A*), we assumed that there was no essential difference in protein function between them. We did not strictly discriminate each pair of paralogs in the following analyses and labeled them “*CpSPCH*”, for example (details in *Materials and Methods*). We were unable to find an FAMA ortholog in *C*. *stagnalis*, but this may have been due to the low-quality assembly for this species (*SI Appendix*, Table S1).

We checked the expression patterns of the *SMF* orthologs identified in *Callitriche* by performing whole-mount in situ hybridization (WISH). As representatives of each lifestyle, we analyzed *C*. *palustris*, the species in which we recently established the experimental system (23), and *C. japonica,* the species that underwent more rounds of amplifying division (Fig. 3). Expression of both *CpSPCH* and *CjSPCH* was observed in the basal regions of developing leaf primordia, but the *CpSPCH* signal was found more toward the apex compared with the *CjSPCH* signal (Fig. 1*C*). Although localization to specific cells was not observed in *SPCH* orthologs, the *CpSPCH* signal was clearly in the adaxial epidermis, where stomata are actively produced (*SI Appendix*, Fig. S4), and probably included stomatal precursor cells in the proliferation zone of this species (23). Both *MUTE* and *FAMA* orthologs were expressed in rounded GMC-like epidermal cells (Fig. 1*C*). Compared to *CpMUTE*, *CpFAMA* was expressed more toward the apex. Furthermore, *CpFAMA-*expressing cells were more widely distributed in larger leaf primordia, with signals also found in young GCs (*SI Appendix*, Fig. S5). *CjMUTE* and *CjFAMA* showed similar expression patterns as the *C. palustris* orthologs, but their signals were more scattered across the primordia (Fig. 1*C*). Overall, these findings suggest that the sequential expression patterns of *SPCH*, *MUTE*, and *FAMA* in the stomatal lineage are conserved in *Callitriche*. We further confirmed that all of the characteristic domains of each SMF protein (34) were identifiable in *Callitriche* orthologs (*SI Appendix*, Fig. S6), implying that the protein function of SMF is also conserved in *Callitriche*.

### Different Expression Patterns of *SPCH* and *MUTE* Underlie the Diversity in Meristemoid Division.

Among the SMF proteins, SPCH and MUTE, transcription factors that maintain (3) and terminate (2) the amplifying division, respectively, are the most likely candidates underlying the interspecific diversity in meristemoid behavior (12, 16). We hypothesized that either different functions or expression patterns of *SPCH* and/or *MUTE* were responsible for the diversity in cell division among *Callitriche* species. Because we did not find any notable substitution in amino acid sequences that might account for the different protein functions among *Callitriche* species (*SI Appendix*, Fig. S6), we focused primarily on the gene expression patterns.

**Fig. 4.**
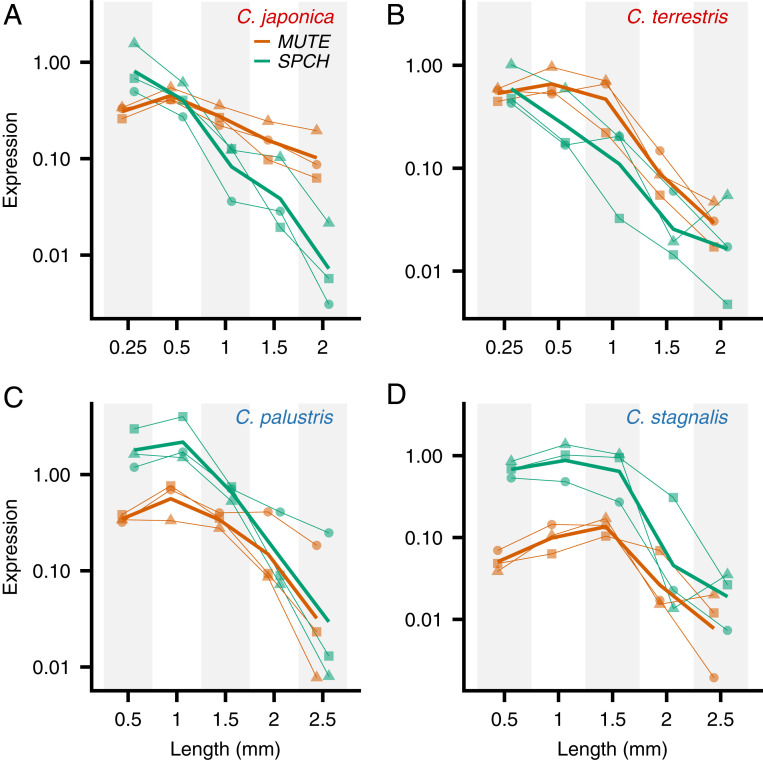
Expression patterns of *SPCH* and *MUTE* orthologs in different stages of leaf development (represented by primordium length) in *Callitriche* species. The expression of each gene was standardized to *SAND1* expression (internal control) and shown in log scale. The data obtained from a single individual are shown by the same symbol for each species and connected with a thin line (*n* = 3; biological replicates). Mean values from the replicates are shown with bold lines. (*A*) *C*. *japonica*. (*B*) *C*. *terrestris*. (*C*) *C*. *palustris*. (*D*) *C*. *stagnalis*.

The expression patterns of *SPCH* and *MUTE* throughout the course of development were analyzed by real-time PCR. We collected leaf primordia at five different developmental stages (defined by size) from healthy growing shoots of each species. The youngest stage tested corresponded to the primordium length at which stomata started to differentiate (Fig. 2*B*; primordium length of 0.5 mm for amphibious species and 0.25 mm for terrestrial species), followed by four further stages in larger primordia. We extracted RNA from each sample and quantified the expression levels of *SPCH* and *MUTE* orthologs. Interestingly, we found two distinct expression patterns related to the lifestyles of the species. In the terrestrial species *C*. *japonica* and *C*. *terrestris*, *SPCH* expression was highest at the earliest developmental stage examined, while *MUTE* expression peaked at later stages (Fig. 4 *A* and *B*). In *C*. *palustris*, however, the expression levels of both genes peaked at the same developmental stage (Fig. 4*C*). A similar trend was observed in the other amphibious species, *C*. *stagnalis* (Fig. 4*D*). This unique pattern in amphibious species can be interpreted thus: *SPCH* and *MUTE* expression levels are synchronous; that is, the time lag between *SPCH* and *MUTE* expression is shorter in amphibious species than in terrestrial species (see Fig. 7).

Most importantly, the observed expression patterns were sufficient to construct a model explaining the molecular basis of meristemoid division diversity in *Callitriche* (see Fig. 7). According to our model, *SPCH*-expressing meristemoids in terrestrial species undergo amplifying divisions until *MUTE* is expressed, as in *Arabidopsis*. In amphibious species, however, *MUTE* is prematurely expressed immediately after *SPCH* expression, leaving no time for the meristemoids to divide; therefore, they differentiate directly into GMCs.

### Differences in the Timing of *SPCH* and *MUTE* Expression Confirmed at the Cellular Level.

**Fig. 5.**
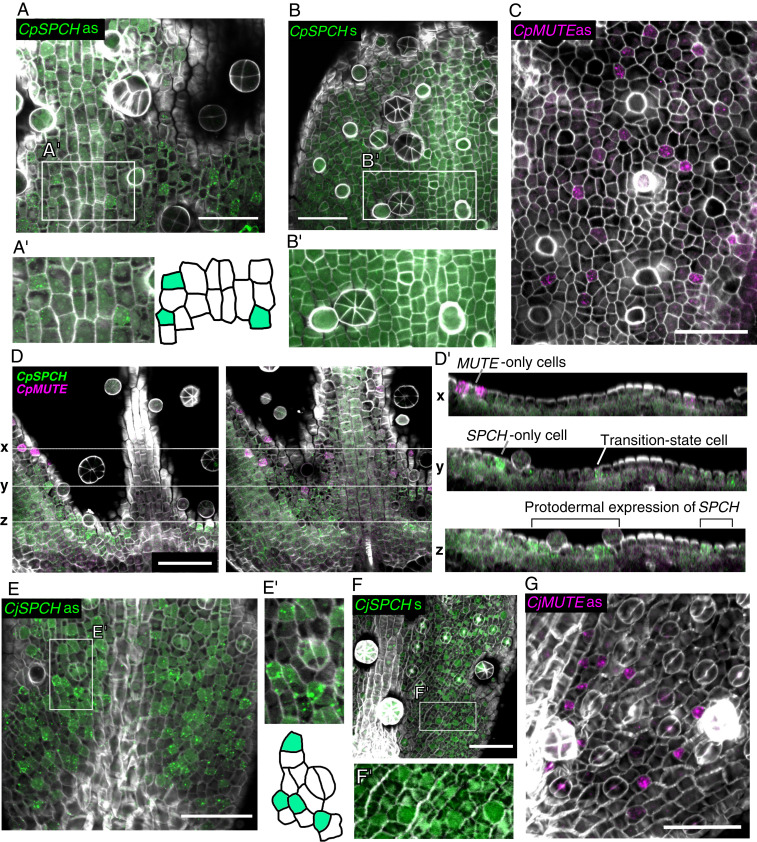
FISH detection of *SPCH* (green) and *MUTE* (magenta) orthologs in *C. palustris* (*A*–*D*) and *C. japonica* (*E*–*G*) leaf primordia; cell walls stained (gray) with Calcofluor. (*A* and *B*) The results from antisense (as) and sense (s) probes of *CpSPCH*. (*A'*) and (*B'*) show close-up views of the white boxed frames in *A* and *B*. (*C*) Results from the antisense probe of *CpMUTE*. Results from the sense probe are shown in *SI Appendix*, Fig. S7*A*. (*D*) Serial optical sections of a *C. palustris* leaf primordium after double-detecting *CpSPCH* and *CpMUTE* with antisense probes. Cross-sectional images were constructed by Fiji and are shown in *D'*. The positions of the three sections (*x*, *y*, and *z*) are shown by the white dotted line. In the cross-sections, a transition-state cell (*y*; Fig. 6*C*), an *SPCH*-only cell (*y*), and *MUTE*-only cells (*x*) are identifiable in the epidermal cell layer. In the basal region of the primordia, *SPCH* is broadly expressed but is still restricted to the epidermal (possibly protodermal) cells (*z*). (*E* and *F*) Results from antisense (as) and sense (s) probes of *CjSPCH*. *E'* and *F'* show close-up views of the white box frames in *E* and *F*. (*G*) Results from the antisense probe of *CjMUTE*. Results from the sense probe are shown in *SI Appendix*, Fig. S7*B*. (Scale bars: 50 μm.)

**Fig. 6.**
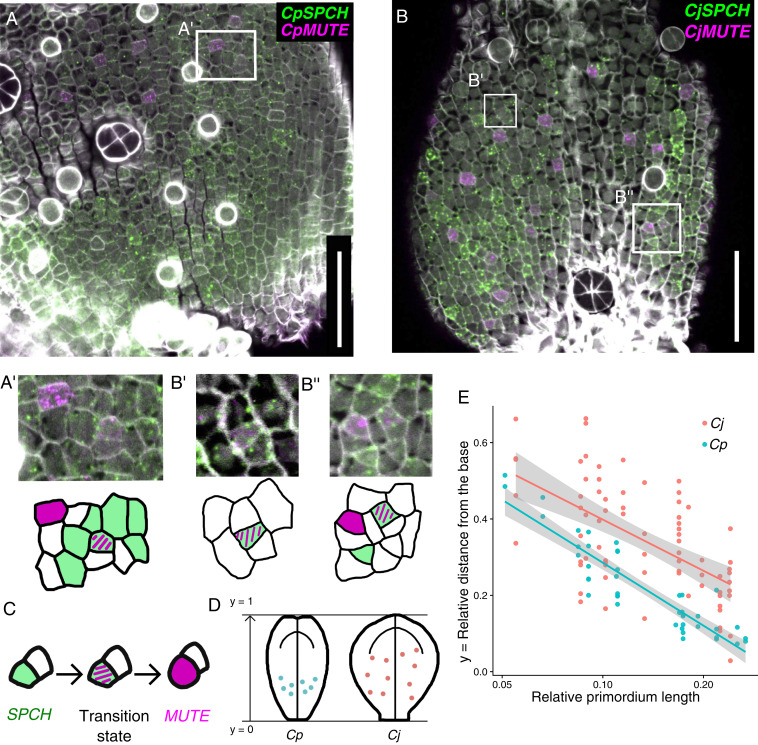
Distribution of transition-state cells, in which *SPCH* and *MUTE* are coexpressed, in *C. japonica* and *C. palustris*. (*A* and *B*) FISH detection of *SPCH* (green) and *MUTE* (magenta) orthologs in *C. palustris* (A; primordium length, 1,137 μm) and *C*. *japonica* (B; primordium length, 352 μm); cell walls stained (gray) with Calcofluor. (*C*) Cells in a transition state showing coexpression of *SPCH* and *MUTE* are identified for each species (*A'*, *B'*, and *B''*; close-up views of the box frames in *A* and *B*). (Scale bars: 50 μm.) (*D* and *E*) Quantification of the positions of cells in a transition state. (*D*) Graphic summary of the results. (*E*) The coordinate position of each cell was calculated along the main axis of the leaf primordium and plotted against the relative primordium length standardized by the length of the mature leaf. Therefore, each dot corresponds to one transition-state cell (*n* = 43 for *C. palustris* and *n* = 73 for *C. japonica*), and each row of dots corresponds to a different leaf primordium (*n* = 13 for each species). Linear regression plots are shown with 95% confidence intervals.

**Fig. 7.**
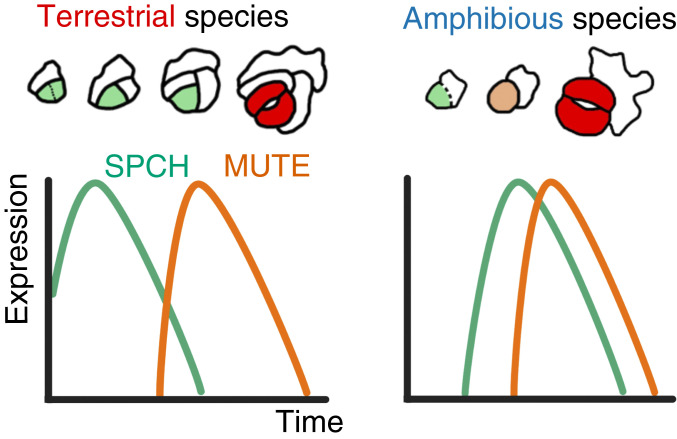
Graphic summary of the results. Meristemoids in the terrestrial species of *Callitriche* (*Left*) undergo a series of amplifying divisions, while those of amphibious species (*Right*) do not divide further but instead differentiate directly into stomata. Different expression patterns of the orthologs of *SPCH*, which maintains division, and *MUTE*, which suppresses *SPCH* activity, underlie this diversity in the behavior of meristemoids. *MUTE* expression is delayed in terrestrial species, but *MUTE* is expressed shortly after the onset of *SPCH* expression in amphibious species; hence, division is terminated early during the development of amphibious taxa.

Although the model that we developed based on the results of the real-time PCR experiments seemed to effectively explain the interspecific variation observed (see Fig. 7), the RNA samples were all extracted from whole leaf primordia, so that the results should be interpreted with caution. Whether individual meristemoids from each species followed the same pattern of gene expression that we observed in the bulk experiment remains to be determined. To analyze the expression patterns at higher resolution, we developed a double-color whole-mount fluorescent in situ hybridization (WM-FISH) system for *Callitriche* species. Combining the WM-FISH system with cell wall staining allowed us to visualize gene expression at cellular-level resolution with a high signal-to-noise ratio. Furthermore, simultaneous detection using fluorescein isothiocyanate (FITC) and digoxigenin (DIG)-labeled probes allowed us to analyze the spatial relationship of the expression domains of two different genes. Using this system, we analyzed the expression patterns of *SPCH* and *MUTE* orthologs in developing leaf primordia of *C*. *palustris* and *C*. *japonica* (Figs. 5 and 6 and *SI Appendix*, Figs. S7 and S8). Using antisense probes, we detected positive FISH signals characterized by aggregated granular dots, which are typical of the tyramide signal amplification (TSA) system (35), for all the genes that we tested (Figs. 5 *A*, *C*, *D*, *E*, and *G* and 6 and *SI Appendix*, Fig. S8). We observed no such specific signals by using sense probes (Fig. 5 *B* and *F* and *SI Appendix*, Fig. S7).

In *C*. *palustris*, *CpSPCH* was expressed widely in the basal region of developing leaf primordia (Figs. 5 *A* and *D* and 6*A* and *SI Appendix*, Fig. S8). The signals were restricted to the adaxial epidermis (Fig. 5*D'*), consistent with the colorimetric WISH result (*SI Appendix*, Fig. S4*B*). In smaller primordia, the expression domain of *CpSPCH* was located more apically (*SI Appendix*, Fig. S8*A*), as also demonstrated by colorimetric WISH (Fig. 1*C* and *SI Appendix*, Fig. S4*A*). This may relate to the slow development of the *CpSPCH* peak in the qPCR analysis (Fig. 4*C*). Through FISH analyses, we were able to detect the localization of *CpSPCH* to specific cells, primarily in apical regions of the expression domain (Fig. 5 *A'* and *D'*). Some of the *CpSPCH*-expressing cells were polygonal in shape, which is typical of early meristemoids. Wider *CpSPCH* expression in the basal region indicates general expression in protodermal cells (Fig. 5*D'*). We assume that after these protodermal cells undergo an entry division in the more apical region of the primordium, *CpSPCH* expression becomes limited to the newly produced meristemoid. This *SPCH* expression pattern has been observed in *Arabidopsis* during the very early stage of leaf development (3). A similar pattern was also reported recently in an *Arabidopsis* line, in which the *SPCH* promoter of tomato (*Solanum lycopersicum*) drove green fluorescent protein (*GFP*) expression (10). These findings support our contention that the expression domain of *SPCH* is conserved in *C*. *palustris*.

We observed clear localization of *CpMUTE* signals to polygonal or rounded cells in the epidermis (Figs. 5 *C* and *D* and 6*A* and *SI Appendix*, Fig. S8*A*). These cells had morphologies typical of late meristemoids and young GMCs, as shown by colorimetric WISH (Fig. 1*C*). Notably, we found a small number of cells expressing both *CpSPCH* and *CpMUTE* (Figs. 5*D'* and 6*A'* and *SI Appendix*, Fig. S8*A'*). *CpMUTE* expression was relatively weak in those cells, which displayed typical meristemoid-like morphology and were found in the border region between the expression domains of *CpSPCH* and *CpMUTE*. These findings indicate that the cells coexpressing *CpSPCH* and *CpMUTE* were meristemoids in a transition state; they had just begun to express *MUTE* while residual *SPCH* expression continued to decline (Fig. 6*C*).

In *C. japonica*, the expression domain of *CjSPCH* was broader than that of *CpSPCH* and extended into the apical region, where the expression was localized to polygonal cells (Figs. 5 *E* and *F* and 6*B* and *SI Appendix*, Fig. S8*B*). This pattern differs slightly from that observed in the colorimetric system (Fig. 1*C*), in which a *CjSPCH* signal was found only in the very basal region of leaf primordia. This indicates that the greater sensitivity of FISH enabled detection of the apical expression of *CjSPCH*. Cells expressing *CjMUTE* were similar in morphology to *CpMUTE*-expressing cells (Fig. 5*G*) but had a wider distribution in leaf primordia (Fig. 6*B* and *SI Appendix*, Fig. S8*B*), which is consistent with the colorimetric WISH results (Fig. 1*C*). As for *C*. *palustris*, we found cells in the putative transition state that coexpressed *CjSPCH* and *CjMUTE* (Fig. 6 *B'* and *B''*). Since the expression domains of *CjSPCH* and *CjMUTE* were wide and overlapped in *C*. *japonica*, the transitioning cells were found not only in the basal region, but also in more apical regions, where mature stomata were located close by (Fig. 6*B''* and *SI Appendix*, Fig. S8 *B* and *B'*). When the relative position of the transitioning cells was plotted against relative primordium length, it was clear that the distribution in *C*. *japonica* was wider, extending to more apical regions than in *C*. *palustris* (Fig. 6 *D* and *E*). Unlike *C*. *palustris*, *C*. *japonica* has a petiole-like region in the basal-most region of the leaf, where little cell division activity was seen (*SI Appendix*, Fig. S8*C*). Even after taking account of the presence of the petiole in *C*. *japonica*, the difference in distribution patterns remained (*SI Appendix*, Fig. S8 *D* and *E*).

In *C*. *palustris*, cell proliferation occurred in the basal region of the leaf blade (23), as in *C*. *japonica* (*SI Appendix*, Fig. S8*C*), and *SPCH* orthologs were expressed in dividing cells in both species. Therefore, the basally biased distribution of transition-state meristemoids in *C*. *palustris* demonstrated that the meristemoids began expressing *CpMUTE* immediately after they had been formed, at a time when they were expressing *CpSPCH* (Fig. 6 *D* and *E*). In *C*. *japonica*, however, some of the transitioning cells were located more apically, indicating that the expression of *CjMUTE* was delayed, as shown in our model (Fig. 7).

Notably, the distributions of transitioning cells in the two species overlapped to some extent in the basal regions (Fig. 6 *D* and *E* and *SI Appendix*, Fig. S8 *D* and *E*). Since some meristemoids directly differentiated into stomata without any amplifying divisions even in *C*. *japonica* (Fig. 2*E* and *SI Appendix*, Fig. S8*B*), we expected to see early expression of *CjMUTE* in some proportion of *C*. *japonica* meristemoids. The overlapping distribution of transitioning cells supported this expectation. In summary, the expression pattern that we inferred from the bulk qPCR experiment was confirmed at the cellular level (Fig. 7).

## Discussion

We found diversity in the behavior of stomatal meristemoids among species of *Callitriche*. The meristemoids in the terrestrial species divided asymmetrically multiple times before differentiating into stomata, while those in the amphibious species skipped these divisions and differentiated directly into stomata. Diversity in meristemoid division is common among different taxa of plants (16, 36); however, the diversity that we describe was unprecedented in that it occurred within a single genus and was correlated with lifestyle.

A previous phylogenetic and ancestral reconstruction study suggested a terrestrial origin of the genus *Callitriche*, with subsequent transition to the amphibious mode and multiple reversal events back to the terrestrial lifestyle (26). Among the species used in our analyses, *C*. *japonica* is believed to be an ancestral terrestrial species; however, *C*. *terrestris* appears to have reverted back from the amphibious mode to a terrestrial lifestyle (26, 27) (Fig. 2*A*). The fact that both species clearly had amplifying cell division indicates that additional meristemoid divisions were either independently lost in an amphibious species or were acquired independently in a terrestrial species. This indicates the presence of adaptive significance of the different meristemoid behaviors in the genus *Callitriche*.

The presence of amplifying divisions is thought to be critical for flexible regulation of stomatal arrangement and leaf size in *Arabidopsis* (37, 38). How might the lack of amplifying divisions in amphibious species of *Callitriche* be advantageous for these plants? The amphibious species do not begin stomatal formation until relatively late in development (Fig. 2 *B* and *C* and *SI Appendix*, Fig. S1 *B* and *C*). In *C*. *palustris*, this occurs when the contour of dimorphic aerial and submerged leaves begins to diverge (23), at ∼500 μm in length. This delayed-onset stomatal differentiation might reflect a “pending state” that should be adaptive in a changing environment; that is, if the shoot tip were submerged at this stage, then leaf primordia would suppress stomatal development, whereas otherwise the leaf primordia would quickly differentiate stomata by the time they were fully expanded. The absence of amplifying divisions might be advantageous for amphibious species because it leads to rapid differentiation of stomata, which offsets the delay caused by the pending state. Note that floating or amphibious species in the genera *Cabomba* (Cabombaceae), *Nymphaea* (Nymphaeaceae), *Potamogeton* (Potamogetonaceae), and *Trapa* (Lythraceae) have been reported to lack amplifying divisions in the stomatal lineage (39–42). The aquatic-to-amphibious genus *Subularia* seems to be the only member of the Brassicaceae that lacks the anisocytic stomatal complex (43), which is typically produced by a series of amplifying divisions (Fig. 1*A*). Although these may reflect only the ancestral state of each genus, it is also plausible that the lack of amplifying division has a general advantage in aquatic environments. Although this hypothesis must be tested in future studies, it is clear that the diversity found in *Callitriche* will open the way for testing the adaptive significance of amplifying divisions.

Our molecular genetic analyses showed that the different expression times of *SPCH* and *MUTE* underlie this intragenic diversity in *Callitriche* (Fig. 7), supporting a previously proposed, but unproven, hypothesis that these two genes are involved in the diversity of meristemoid behavior (12, 16). Further verification is needed, but we propose that this change in the temporal regulation of key stomatal transcription factors is responsible for the evolution of the diversity in meristemoid behavior that we described for *Callitriche*. Because of the simplicity of the model, we propose that the same mechanism may underlie the diversity found in other lineages, such as early divergent angiosperms in the basal ANITA clade, which appear to have functional SMF proteins but lack amplifying divisions (17, 42). Further genetic analyses of *Callitriche* are needed to determine the direct mechanism accounting for the interspecific difference in the timing of *SPCH* and *MUTE* expression. The rapidly growing understanding of the regulation and function of the two genes in the model species *Arabidopsis* (44–46) should greatly aid in the challenging future analyses of the nonmodel species of *Callitriche*.

## Materials and Methods

### Plant Materials and Culture Conditions.

The *Callitriche* plants used in this study were originally collected from their wild habitats in Japan and maintained in our laboratory. We collected *C*. *stagnalis*, *C*. *terrestris*, and *C*. *japonica* specimens in Ibaraki Prefecture, Hyogo Prefecture, and Kanagawa Prefecture, respectively, and included them in our analysis together with the previously described *C*. *palustris* NH-1 strain (23). All plants were maintained in a growth chamber at 23 °C under long-day conditions (16 h light/8 h darkness), with a light intensity of 60 μmol photons m^−2^ s^−1^. The specimens used in the experiments were transplanted aseptically to plant boxes containing autoclaved soil (Aqua Soil Amazonia; Aqua Design Amano) and grown under the conditions described above for at least 2 wk (after which sufficient numbers of new shoots and leaves had been formed). For *C. palustris*, *C. terrestris*, and *C. japonica,* the single plant strain of each species collected from their wild habitats was selfed for at least two generations to produce more stable and homozygous lines. In the subsequent experiments, three siblings from one established strain were analyzed and used as the three biological replicates (Figs. 3 *E* and *F* and 4). As *C. stagnalis* did not produce seeds under our culture conditions, asexually propagated wild strains originating from the same locality were used for the experiments.

### Observation of Epidermal Cells.

Shoots and leaves were immersed in formalin-acetic acid-alcohol (FAA) fixative (10% formalin, 5% acetic acid, 50% ethanol [vol/vol]). The fixed samples were then cleared using methods based on either thiodiethanol (TDE) (47) or ClearSee (48). For observation by differential interference contrast microscopy (DM4500; Leica Microsystems), we first transferred the leaf samples into an ethanol series and then treated them with TDE for >1 h before mounting. Smaller samples were treated with 1% Calcofluor White (Sigma-Aldrich) in ClearSee for 2 d to stain cell walls before observation by confocal microscopy (FV10i; Olympus) with UV excitation. Captured images were analyzed using Fiji v1.0 (49). The stomatal index (Fig. 2*B* and *SI Appendix*, Fig. S1 *A*, *B*, and *G*) was calculated based on multiple images of the epidermis captured in at least three different fields of view in the apical halves of the leaf primordia.

### Time-Lapse Observation of the Epidermis.

A dental impression medium-based method (37, 50) was used for time-lapse observations of the epidermis. Leaf surfaces were covered with freshly prepared dental impression medium (Provil novo Light; Kulzer) to make the molds. This procedure was repeated every 1 to 3 d on each leaf to obtain a series of molds throughout the course of leaf development. Finally, we obtained time-lapse epidermal impressions by applying nail polish (AC Quick-Dry Topcoat; Do-Best, Inc.) to each mold, which was then peeled off after the polish had dried. The epidermal impressions were directly mounted onto glass slides and observed by light microscopy (DM4500; Leica Microsystems).

### De Novo Transcriptome Assembly.

To reconstruct the transcriptome, we cultured shoots from single individuals of *C*. *stagnalis* and *C*. *japonica* under either aseptic aerial or submerged conditions. RNA was extracted from whole plant bodies using the RNeasy Plant Mini Kit (QIAGEN) with on-column applications of RNase-free DNase (QIAGEN). The quality of the RNA was measured using the Agilent 2100 Bioanalyzer system with the RNA6000 nano kit; quantities were measured by Qubit fluorometric quantitation (Thermo Fisher Scientific). The libraries for Illumina sequencing were prepared with a stranded mRNA-Seq kit (KAPA Biosystems) using a protocol optimized for a 300- to 400-bp insert size. We sequenced the libraries using the Illumina HiSeq 1500 sequencing system in rapid-run mode and obtained 150-bp paired-end reads for each sample. The raw reads were deposited in the DNA Data Bank of Japan (DDBJ) Sequence Read Archive (DRA; accession nos. DRX227815 [*C. stagnalis*] and DRX227816 [*C. japonica*]). The strategy of de novo transcriptome assembly and decontamination followed that of our previous study (31). We used trimmomatic v0.36 (51), Trinity v2.2.0 (52), rnammer v1.2.1 (53), Corset v1.07 (54), and TransDecoder v3.0.0 software. We also used assemblies of *C*. *palustris* and *C*. *terrestris* that had been reconstructed previously (31). The statistical parameters of the assemblies are listed in *SI Appendix*, Table S1. Assembly sequences have been deposited in figshare (http://dx.doi.org/10.6084/m9.figshare.13450931).

### Identification of SMF Orthologs in *Callitriche* Species.

The transcriptome of each *Callitriche* species was searched for SMF orthologs using BLASTp (protein-protein BLAST). To construct a protein phylogenetic tree, we also conducted BLASTp searches against public protein databases and obtained putative SMF orthologs from other species. The sequences retrieved were aligned using MAFFT v7.453 (55). After trimming nonhomologous regions using trimAL v1.4 (56), we constructed a maximum likelihood tree using RAxML v8.2.12 (57) with 1,000 bootstraps (Fig. 1*B*).

### Quantification of Gene Expression by Real-Time PCR.

Total RNA was extracted from leaf primordium samples of *Callitriche* species using the FastGene RNA Premium Kit (Nippon Genetics). Each sample comprised >10 leaf primordia of approximately the same length. The extracted RNA was then used for reverse transcription with the PrimeScript RT Reagent Kit (TaKaRa Bio) to synthesize cDNA. Real-time PCR was performed using Thunderbird SYBR qPCR Mix (Toyobo) on a Roche LightCycler 480 II PCR platform. We used the *SAND1* gene, which shows stable expression in multiple plant species (58, 59), as an internal control. We also confirmed that *CpSAND1* was stably expressed in all tissues and conditions in our RNA-Seq data (31) (*SI Appendix*, Fig. S3*A*). We quantified the expression of *SPCH* and *MUTE* orthologs in the three biological replicates described above using the comparative CT method (Fig. 4). The results from three technical replicates were averaged and are shown in Fig. 4. The primers used for real-time PCR are listed in *SI Appendix*, Table S2.

For *C. palustris*, we designed primers based on the sequence of one of the two putative paralogs of *SPCH* and *MUTE*. However, considering the high sequence similarity and highly similar expression patterns of the two paralogs (*SI Appendix*, Fig. S3), we did not design primers that amplified each paralog specifically. We assumed that our primers amplified both paralog and thus labeled the results as “*CpSPCH*”, for example.

### WISH.

We used colorimetric in situ hybridization to detect stomata-related genes in whole-mount leaf primordia. The whole-length coding regions of the target genes (*CpSPCH A*, *CpMUTE A*, *CpFAMA A*, *CjSPCH*, *CjMUTE*, and *CjFAMA*) were PCR-amplified from the cDNA that we had synthesized previously (as explained above) using the primers listed in *SI Appendix*, Table S2. The amplified fragments were cloned into the pZErO-2 vector (Thermo Fisher Scientific). After amplification of the template with M13F and M13R primers, we used either SP6 or T7 polymerases (Roche) and the DIG RNA Labeling Kit (Roche) for reverse transcription to synthesize DIG-labeled probes. Using these probes, we performed in situ hybridization as described previously (23), using a modification of the cell wall enzyme treatment (1 h at 37 °C). Samples were visualized by light microscopy (DM4500; Leica Microsystems). For *CpSPCH*, the samples were embedded in Technovit 7100 (Kulzer) and sliced into 15-μm-thick sections using a HM360 rotary microtome (Thermo Fisher Scientific) to examine the signal localization pattern (*SI Appendix*, Fig. S4*B*). Information on the cloned sequences was deposited in the DDBJ (accession nos. LC565133 to LC565137 and LC596432).

For *C. palustris*, we synthesized probes using one of the putative paralogs for each *SMF* gene as in our real-time PCR analysis. Because of the high similarity between each pair of paralogs, we anticipated a fair amount of cross-hybridization between these paralogs (e.g., *MUTE*; *SI Appendix*, Fig. S3*B*). Therefore, we did not strictly discriminate one paralog from the other in our in situ hybridization experiments and thus labeled the results as “*CpSPCH*”, for example.

### FISH.

To detect gene expression at higher resolution, we applied FISH to whole-mount leaf primordia of the *Callitriche* species. The method followed the previously described protocol for the *Arabidopsis* inflorescence meristem (35), with some modifications. DIG- and FITC-labeled RNA probes of *Callitriche MUTE* and *SPCH* were synthesized with the DIG RNA Labeling and Fluorescein RNA Labeling mixes (Roche), as described above, using the same plasmids. To detect the DIG probes, we used the mouse anti-digoxigenin antibody IgG1k (Roche) and goat anti-mouse antibody in a tyramide amplification system (Alexa Fluor 488 Tyramide SuperBoost Kit, goat anti-mouse IgG; Invitrogen). We detected the FITC probes using rabbit anti-FITC polyclonal antibody (Invitrogen) and goat anti-rabbit antibody in a tyramide amplification system (Alexa Fluor 594 Tyramide SuperBoost Kit, goat anti-rabbit IgG; Invitrogen). Probe hybridization, antibody reactions, and tyramide signal amplification (TSA) were performed as described previously (35). We successfully obtained specific granular-like signals, which are typical of the TSA system (35). The samples were then treated with 1% Calcofluor White in ClearSee solution for 2 d to stain the cell walls before visualization by confocal microscopy (Fv10i; Olympus). Using our *SPCH* and *MUTE* double-detection procedure, we obtained signals in *C*. *palustris* clearly showing identical tendencies in the combination of FITC-labeled *CpSPCH* and DIG-labeled *CpMUTE* probes and in the combination of DIG-*CpSPCH* and FITC-*CpMUTE* probes. The results were combined for further analyses (Fig. 6*E* and *SI Appendix*, Fig. S8*E*). In *C*. *japonica*, however, the FITC-*CjSPCH* probe did not produce specific signals (for an unknown reason); thus, we used only the results from DIG-*CjSPCH* and FITC-*CjMUTE* probes in our analysis.

### Detection of the Cell Proliferation Zone in *C. japonica*.

The cell proliferation zones in the leaf primordia of *C*. *japonica* were visualized using a 5-ethynyl-2′-deoxyuridine (EdU)-based procedure (*SI Appendix*, Fig. S8*C*). We used the Click-iT EdU Alexa Fluor 488 Imaging Kit (Thermo Fisher Scientific), and followed previously described protocols (23, 60) to incorporate EdU and detect the fluorescence signal. To incorporate EdU, we incubated the samples for 10 h under the normal culture conditions described above. After signal detection, the samples were visualized by fluorescence microscopy (DM4500; Leica Microsystems).

## Supplementary Material

Supplementary File

## Data Availability

RNA-Seq reads have been deposited in the DDBJ DRA (accession nos. DRX227815 and DRX227816). Cloned sequences have been deposited in the DDBJ (accession nos. LC565133 to LC565137, and LC596432). Assembly sequences have been deposited in Figshare (http://dx.doi.org/10.6084/m9.figshare.13450931).
