# Supplementary material for: The diversity of stomatal development regulation in Callitriche is related to the intrageneric diversity in lifestyles
Source: Proc Natl Acad Sci U S A. 2021 Mar 29;118(14):e2026351118. doi: 10.1073/pnas.2026351118 (PMC8040647; doi:10.1073/pnas.2026351118)
Supplement: Supplementary File [file pnas.2026351118.sapp.pdf]

PNAS
www.pnas.org

Supplementary Information for
The diversity of stomatal development regulation in *Callitriche* is related to the intrageneric diversity in lifestyles.

Yuki Doll, Hiroyuki Koga, and Hirokazu Tsukaya

Hiroyuki Koga
Email: hiro1224koga@gmail.com

This PDF file includes:

Figures S1 to S8
Tables S1 to S2
SI Reference

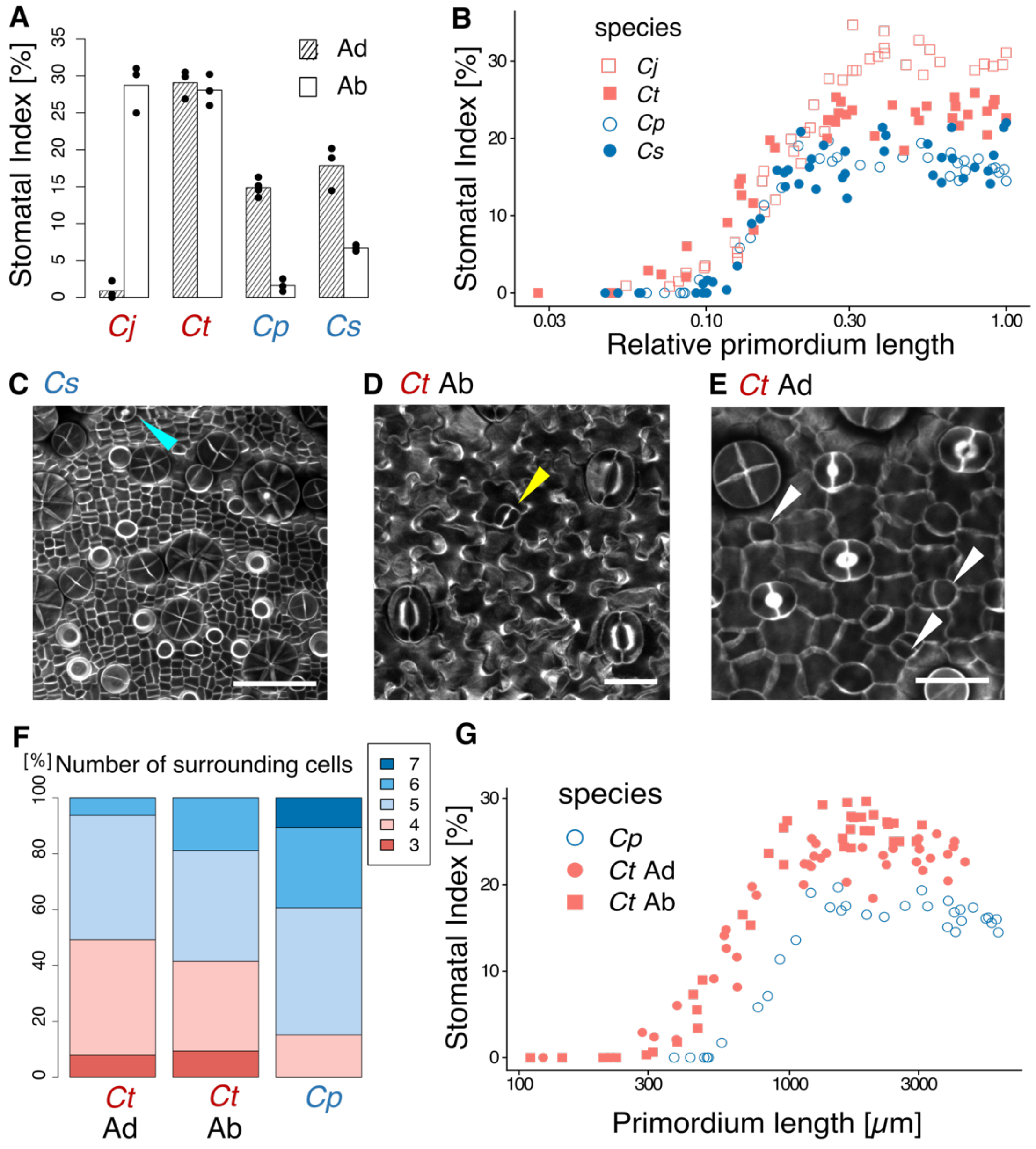

Fig. S1. Content related to Fig. 2. (A) Distribution of stomata on both sides of the leaf in *Callitriche* species quantified by the stomatal index (SI). Each dot corresponds to one leaf primordium (n = 3 or 4). (B) Quantification of the SI through the developmental period; leaf primordium length is standardised by the maximum primordium length for each species. Each dot corresponds to one leaf primordium. (C) Adaxial epidermis of a *C. stagnalis* leaf primordium (length = 637 μm). Blue arrowhead indicates a newly formed stoma. Scale bar = 50 μm. (D) Abaxial epidermis of a *C. terrestris* leaf (length = 2,280 μm). Yellow arrowhead indicates a small stoma surrounded by three pavement cells that are likely to have undergone a series of amplifying divisions. Scale bar = 20 μm. (E) Adaxial epidermis of *C. terrestris* leaf primordia (length = 717 μm). White arrowheads indicate meristemoid-like cells. Scale bar = 20 μm. (F,G) Analyses of the adaxial stomata

in *C. terrestris*. (F) Number of pavement cells surrounding a stoma. (G) Change in SI throughout the course of development.

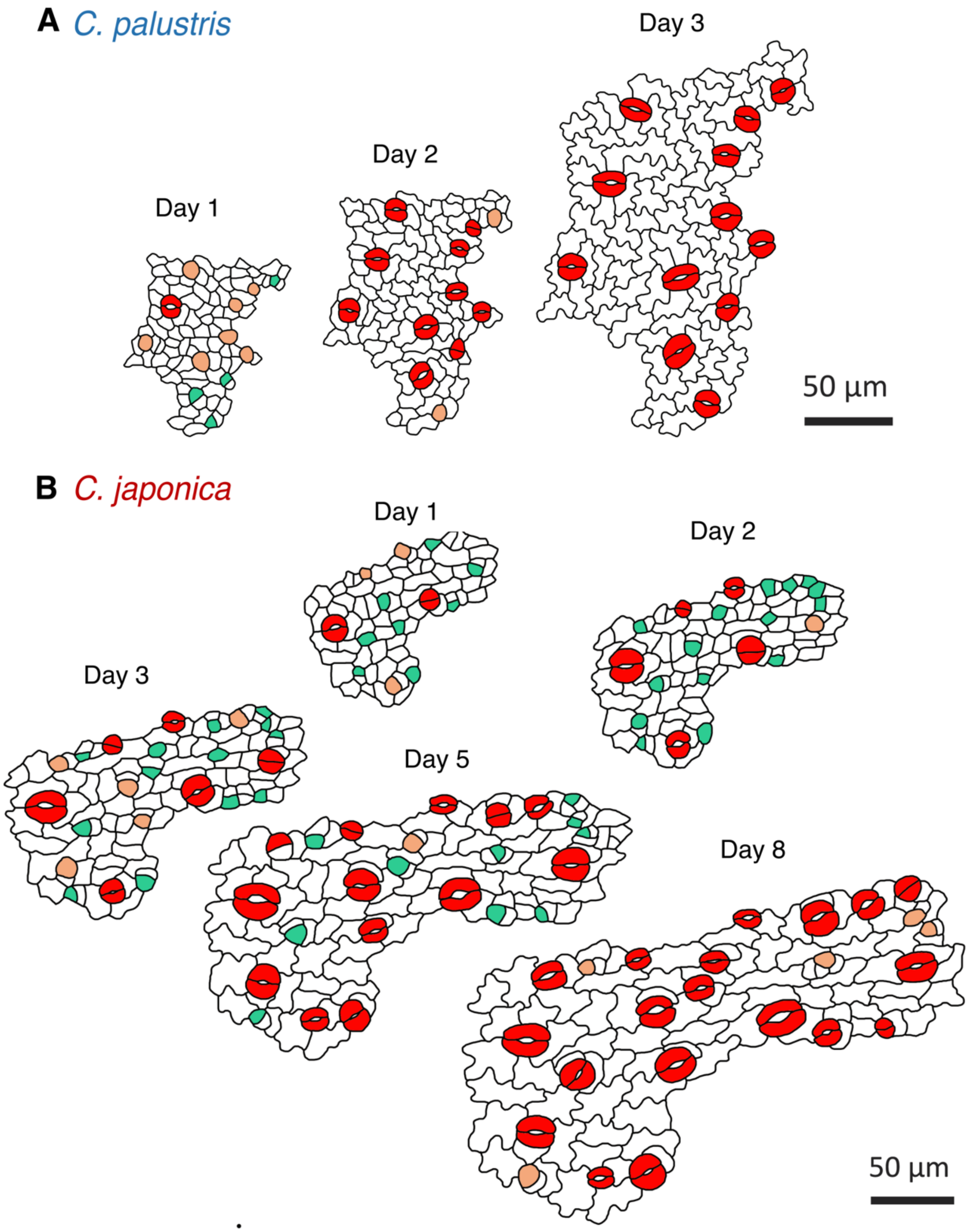

Fig. S2. Content related to Fig. 3. Examples of time-lapse analyses of epidermal impressions in *Callitriche palustris* (A) and *C. japonica* (B). Traces of cell contours are shown with the colours corresponding to the different stages of stomatal development (Fig. 1A).

A

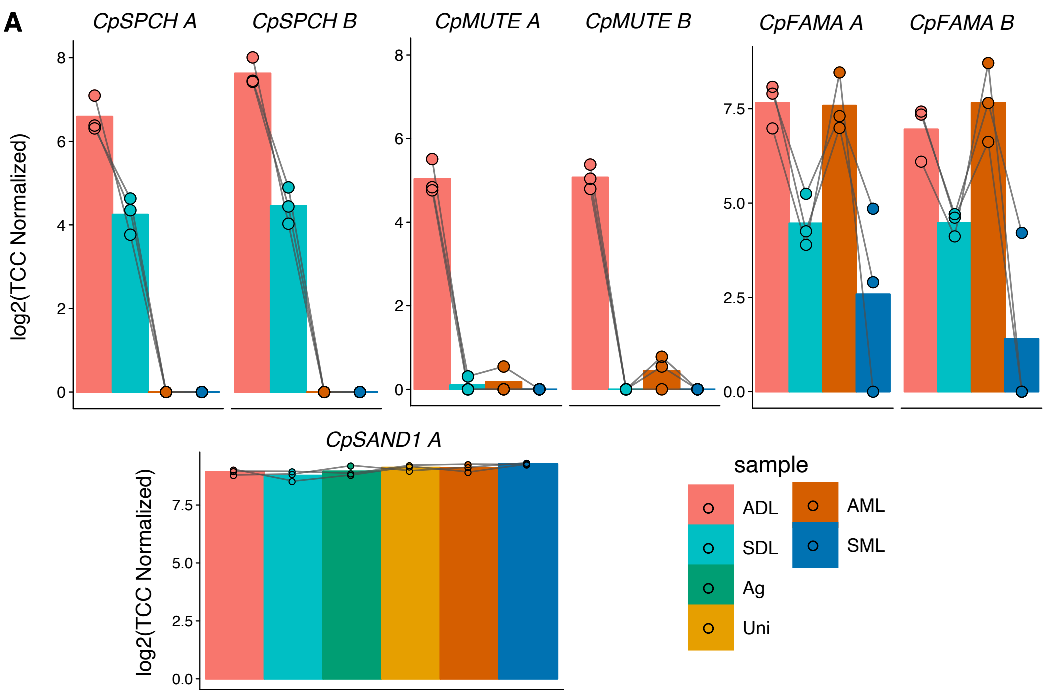

B

```
           1                                                                                82
MUTEA_cDNA GGTAAAACAAGAATTTCCTAAGCTCAATTCTTTTCGTCATCATAATAAGTAAGGTTGAGAATATCATGTCTCACATAGCTGT
MUTEB_cDNA GGTAAAACAAGAATTTCCTAAGCTCAACTCTTGTTGCCATCACAATAAGTAACGGTTGAGAAA--ATGTCTCACATAGCTGT

..........................................................................................

           83                                                                              164
MUTEA_cDNA GGAGAGGAACAGGAGAAGACAGATGAACGAGTACCTCAAGGTTTTACGATCACTGACTCCATGTTTCTACATCAAACGGGGA
MUTEB_cDNA TGAGAGGAACAGAAGAAGGCAGATGAACGAGTACCTCAAGGTTTTACGATCATTAACTCCTTGTTTCTACATCAAACGGGGT

..........................................................................................

           165                                                                             246
MUTEA_cDNA GATCAAGCATCAATTATTGGTGGTGTGGTGGAATTCATCAAGGAGTTACACCAAATCCTACAATCTCTGGAGGCTAAAAAAC
MUTEB_cDNA GATCAAGCATCAATTATAGGTGGGGTGGTGGAGTTCATCAAGGAGTTGCACCAAATCCTACAATCTTTGGAGGCTAAAAAAC

..........................................................................................

           247                                                                             328
MUTEA_cDNA GAAGGAAGAGCATAAGCCCCAGCCCTAGTCCAAGGCCATTGCAACTTACAAGCCCTCTGCCAGATAGCCTGTTTTTAGACAT
MUTEB_cDNA GAAGGAAGAGCATAAGTCCTACCCCTAGTCCAAGGCCATTGCAACTTATAAGTCCTCGAACAGATAGCTCATTTATAGACAT

..........................................................................................

           329                                                                             410
MUTEA_cDNA GAACAACAATTTCAAGGAACTAGGCGCGTCATGCAACTCTCCCATTGCAGATGTTGAAGCCAAAATTTCTGGGTCAAACGTT
MUTEB_cDNA GAATAACAATTTCAAGGAGCTAGGAGCATCCTGCAACTCCCCCATTGCAGATGTTGAAGCCAAGATTTCTGGATCAAACGTT

..........................................................................................

           411                                                                             492
MUTEA_cDNA CTATTAAGGACTATATCGAGGCGGATCCCAGGTCAAATTGTCAAGATAATCAGTGTGTTGGAGAATCTCTCGTTTGAGATTC
MUTEB_cDNA CTGCTAAGGACCATATCTAGGCGAATCCCAGGTCAGATTGTGAAGATTATCAGTGTGTTGGAGAATCTTTCGTTTGAGATTC

..........................................................................................

           493                                                                             574
MUTEA_cDNA TTCACTTGAACATCAGTAGCATGGAAGACACTGTGTTGTATTCCTTTGTCATCAAGATAGGATTGGAGTGTCAAATAAGCTT
MUTEB_cDNA TTCACTTGAACATCAGTAGCATGGAAGATACTGTTCTATACTCTTTCGTGATCAAGATAGGGTTGGAATGTCAAATAAGCTT

..........................................................................................

           575                                                                             656
MUTEA_cDNA GGAGGAACTGGCTAACGAAGTTCAGCAAAGTTTCTACACTGGTGCAACTTGTATCAAACAAGACATATAACTGCTGTTTAGA
MUTEB_cDNA GGAGGAACTGGCTAACGAAGTTCAGCAAAGTTTCTACACCGGAGCAACTTGTATCAAACAAGAC--ATGATTGTTGTTTAGA

..........................................................................................

           657                                                                             738
MUTEA_cDNA ACTTTTGTCATAAATGGAGTTTTTCGTGAGTCTAGAAAGTATCGCCGATTCAAAATAATTATACCGATAATTAGAACAAATC
MUTEB_cDNA ACTTTTGTTATACATGGAG-ATTTCGCAAGGCTAAAAGGAA--ATTGTTTCGAAATTGGTATACGGATAATTAGAACAAATC

..........................................................................................

           739                                 775
MUTEA_cDNA GTTCTGGAAA-CATACATTGAAAAATCATGCTCTCTG
```

Fig. S3. Content related to Fig. 1. Comparison of the two paralogues of each *SMF* gene in *C. palustris*. (A) Expression of each *SMF* paralogue and *SAND1* (qPCR internal control) under various conditions (ADL, developing leaf primordia in aerial shoots; SDL, developing leaf primordia in submerged shoots; AML, mature leaves in aerial shoots; SML, mature leaves in submerged shoots; Ag, $AgNO_3$-treated developing leaf primordia in submerged condition; Uni, Uniconazole-treated developing leaf primordia in submerged condition), based on the RNA-Seq data from our study (1). (B) Comparison of the nucleotide sequences of the two *MUTE* orthologues in *C. palustris*. Mismatched or deletion sites are shown in red, and the primer binding sites for the real-time PCR analysis are shown by the green bars.

A B

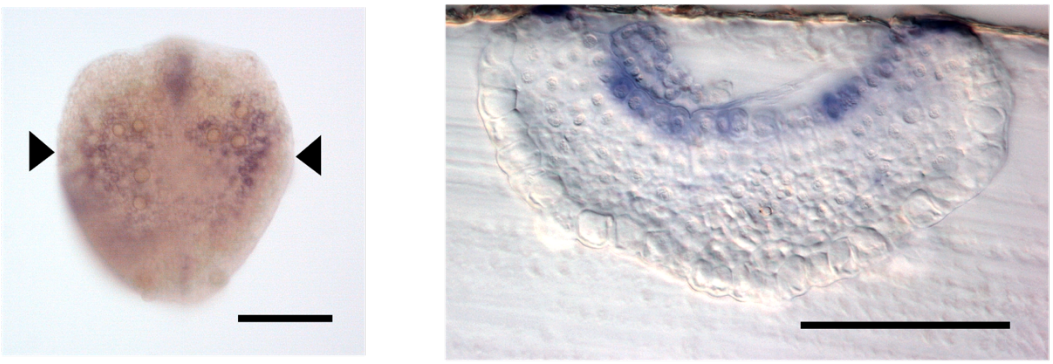

Fig. S4 Content related to Fig. 1. Expression of *CpSPCH* in a young leaf primordium. The primordium shown in (A) was cut at the position indicated by the black arrowheads to make a cross-section (B). Bars = 100 µm.

A
B
C
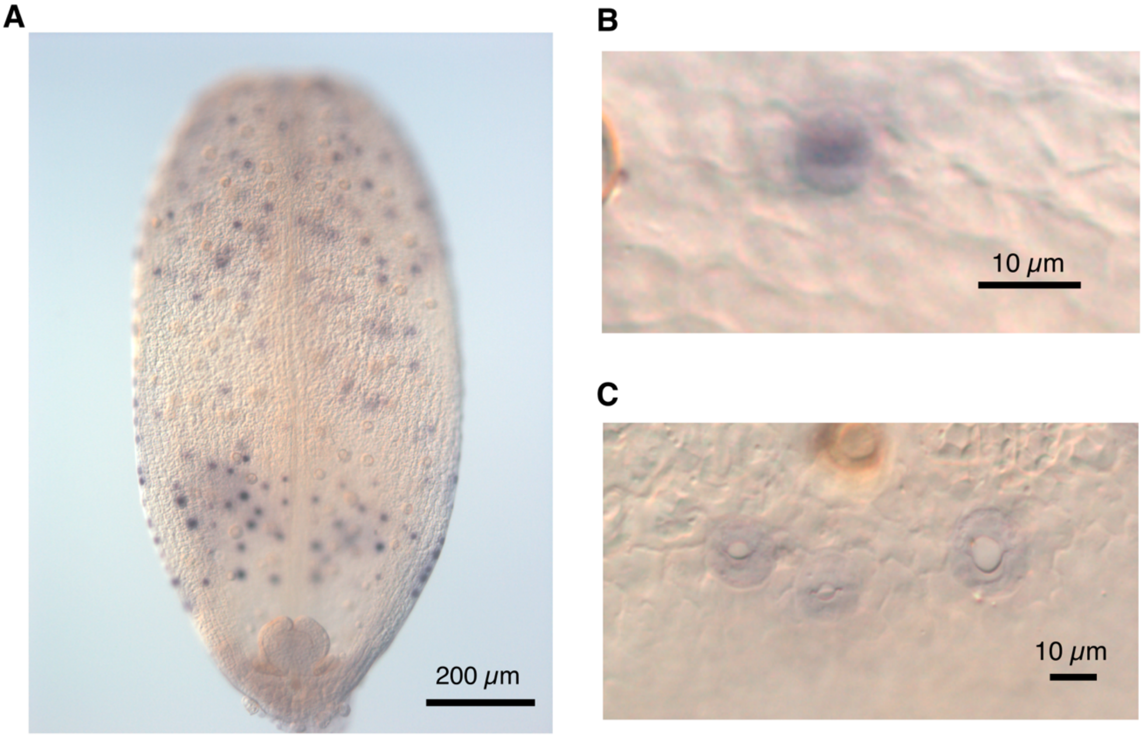

Fig. S5 Content related to Fig. 1. Expression of *CpFAMA* in larger leaf primordia. *CpFAMA*-positive cells are scattered over the whole primordium (A). Signals were detected in late guard mother cells (GMCs) (B) and guard cells (GCs) (C).

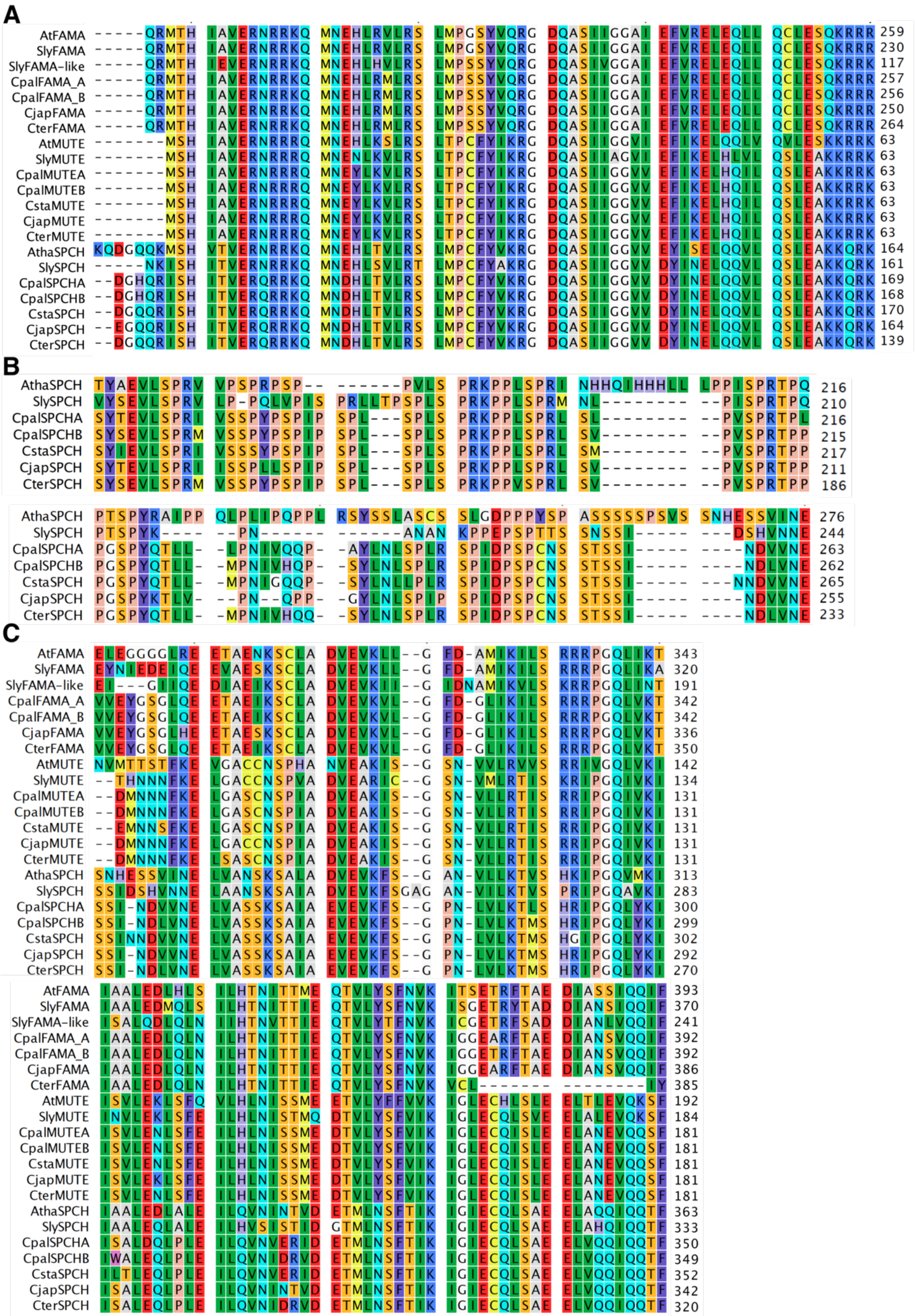

Fig. S6 Content related to Fig. 1. Conserved domains of key transcription factors (SMF proteins). Orthologues from Arabidopsis, tomato, and *Callitriche* species were aligned

with MAFFT software, and visualised with CLC Sequence Viewer (v 7.8.1; QIAGEN). (A) bHLH domain. (B) MAP kinase target domain in *SPCH*. (C) SMF domain.

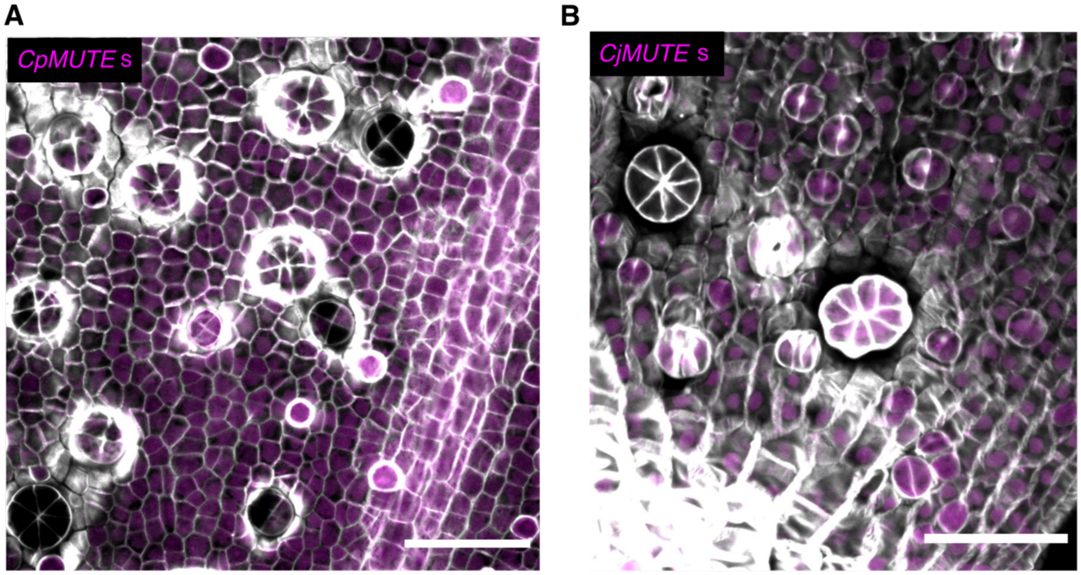

Fig. S7 Content related to Fig. 5. FISH results produced by sense probes of *CpMUTE* (A) and *CjMUTE* (B). Bars = 50 μm

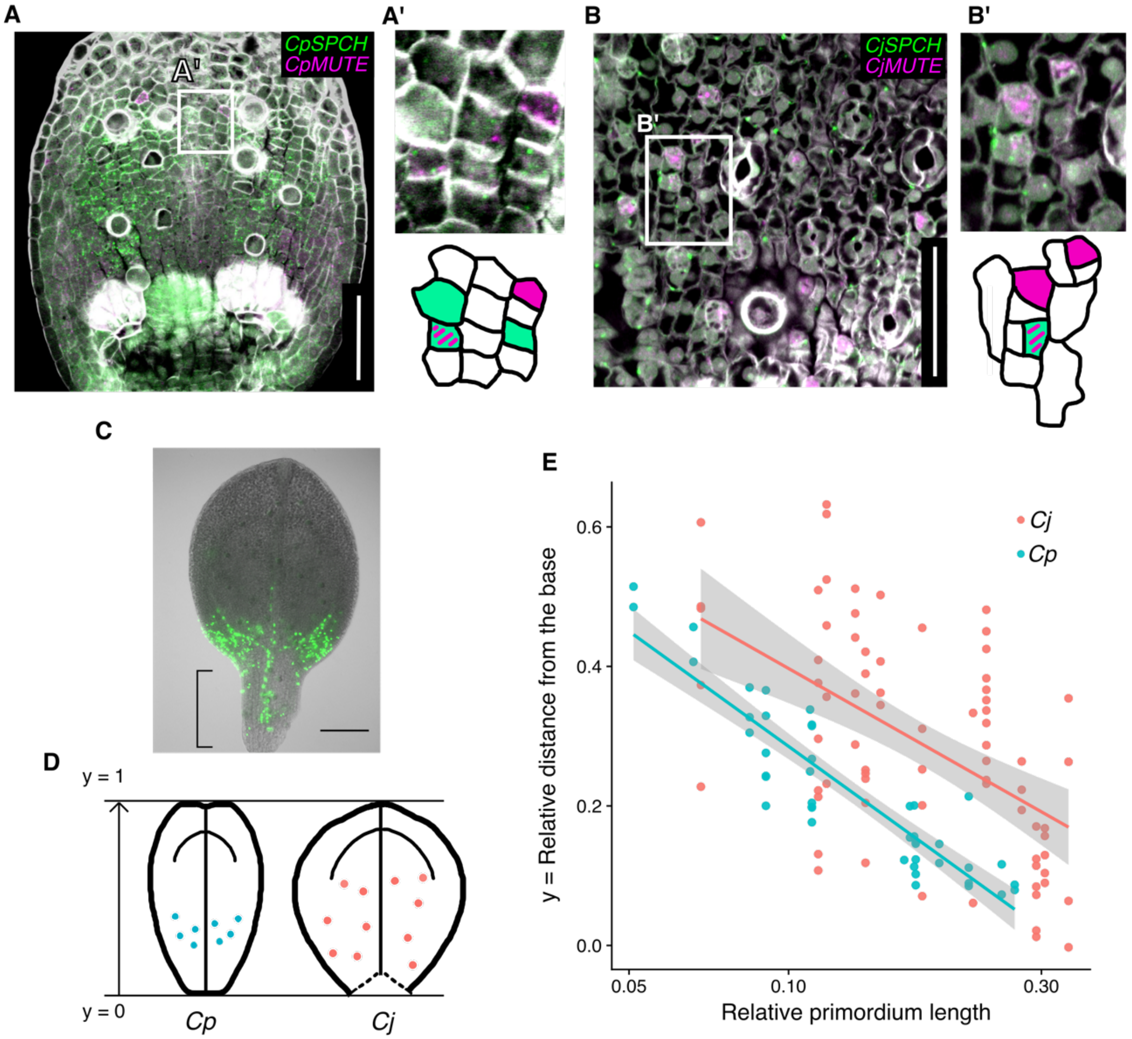

Fig. S8 Content related to Fig. 6. Supplementary information for the fluorescent *in situ* hybridisation (FISH) double detection of *SPCH* (green) and *MUTE* (magenta). (A,B) Additional images of FISH results for *Callitriche palustris* (A; primordium length: 352 µm) and *C. japonica* (B; primordium length: 625 µm). Cells in the transition state were also identifiable in these samples (A', B'). Scale bars = 50 µm. (C) Visualisation of the cell proliferation zone in a *C. japonica* leaf primordium through 5-ethynyl-2′-deoxyuridine (EdU) incorporation. Cells in the S-phase are identified by green fluorescence (see Materials and Methods for details). A petiole region with low proliferation activity is evident in the basal-most region of the primordium (black bracket). Scale bar = 200 µm. (D,E) Analysis of transition state cells in *C. japonica* accounting for the presence of the petiole. (D) Summary of results. The relative distance from the base in *C. japonica*, and the relative primordium length, were recalculated after removing the petiole region, following which the analyses in Fig. 6E were performed again.

Table S1. Transcriptome status of four *Callitriche* species

Species		*C. palustris*	*C. terrsstris*	*C. japonica*	*C. stagnalis*
# of gene		145,929	74,145	87,890	64,356
# of transcript		243,784	130,314	163,350	224,939
Total base		99,628,636	62,970,934	67,484,184	46,194,405
N50		1,040	1,229	1,329	1,156
Mean		721	757	768	718
BUSCO v4 (eudicotyledon)	Complete	88.5%	88.3%	88.7%	75.4%
	Fragment	4.5%	3.8%	4.7%	7.5%
	Missing	7.0%	7.9%	6.6%	17.1%
# of CDS gene		80,437	40,731	43,501	38,338
Source		(1)	(1)	this study	this study

Table S2. List of the primers used in this study.

**Name**	**Sequence**
**Primers for cloning**	
CpFAMA_A_F	GTATGAATGTCAAACCAATCATGTGTC
CpFAMA_A_R	ATACATCACGAAAACTAATACGTGCTC
CpSPCH_A_F	TACATACACCTTTACAAAATCCTCACA
CpSPCH_A_R	CAATATCCTGAGAAAATGGAAATAAAA
CpMUTE_A_F	TTCCTAAGCTCAATTCTTTTCGTCATC
CpMUTE_A_R	ATTTTGAATCGGCGATACTTTCTAGAC
CjFAMA_F	TTCTTGTCTTTTTCTTGAAATTACTCC
CjFAMA_R	CGAATAAAACTGCATTTACATATTCAAG
CjSPCH_F	TTTCTGAAATAGTGTTGATGGCGATAG
CjSPCH_R	AAACATGGCTTCAAGAATGAGAGTTAG
CjMUTE_F	ACGCAGCAACTAAAATAAAATTGGC
CjMUTE_R	AAACGTATTTCTTCGTAGCCTTGTG
**Primers for Real-Time PCR**	
q_CpSPCH_A_F	TCGATCTCTCATGCCTTGC
q_CpSPCH_A_R	GGACTTTCGTTGCTTTTTGG
q_CpMUTE_A_F	CAGGAGAAGACAGATGAACGAG
q_CpMUTE_A_R	TCCAGAGATTGTAGGATTTGGTG
q_CpSAND1_A_F	TGGCATTTGTTTACGGCTATG
q_CpSAND1_A_R	AGGCATCTGAACTCGCTGTG
q_CjSPCH_F	TCTCACTGTTCTTCGCTCTC
q_CjSPCH_R	GGACTTGTTGGAGTTCGTTG
q_CjMUTE_F	GCGGATCCCAGGTCAGATTG
q_CjMUTE_R	TCCAAGCTTATCTGGCACTCC
q_CjSAND1_F	TGCCAAGATACAGTCCCATGG
q_CjSAND1_R	GGCATCTGAACTCGTTGTAAGC
q_CtSPCH_F	CTTCGATCTCTCATGCCTTGC
q_CtSPCH_R	AGCTTCGAGGGATTGGAGTAC
q_CtMUTE_F	AGCATGGAAGACACTGTGTTG
q_CtMUTE_R	GTTAGCCAGTTCCTCCAAGC
q_CtSAND1_F	CCAAGATACAGTGCCATGGC
q_CtSAND1_R	GAAGGCATCTGAACTCGCTG
q_CsSPCH_F	TGGAGCTCGTTGATGTAGTCG
q_CsSPCH_R	ACTGTTGAGAGGCAAAGGAGG
q_CsMUTE_F	TCAGTGTGTTGGAGAATCTCTCG
q_CsMUTE_R	GCTGAACTTCGTTAGCCAGTTC
q_CsSAND1_F	CAATCCGAATCCTGCTGTCC
q_CsSAND1_R	TGCTTGCCAAGATACAGTGC

SI Reference

1. H. Koga, M. Kojima, Y. Takebayashi, H. Sakakibara, H. Tsukaya, The molecular framework of heterophylly in *Callitriche palustris* L. differs from that in other amphibious plants. bioRxiv (2020) https:/doi.org/10.1101/2020.12.19.423571.
